# What are the cardiovascular responses during blood flow-restricted resistance exercise?

**DOI:** 10.3389/fphys.2024.1417855

**Published:** 2024-06-20

**Authors:** James O. Suggitt, Brock E. Eaves, Marty D. Spranger

**Affiliations:** Department of Physiology, Michigan State University, East Lansing, MI, United States

**Keywords:** blood flow-restricted exercise, kaatsu training, occlusion training, cardiovascular, hemodynamics, blood pressure

## 1 Introduction

Blood flow restriction resistance exercise (BFR-RE) is a rapidly expanding exercise methodology demonstrated to increase muscle mass and performance (Loenneke et al., 2012). While traditional recommendations for muscle hypertrophy suggest resistance training above 70% of the one repetition maximum (1RM) load (American College of Sports Medicine, 2009), BFR-RE purports similar increases in muscle strength and size with as little as 20% of the 1RM (Abe et al., 2005). This is accomplished by the restriction of blood flow to the exercising muscle through the application of a pressure device, typically a pneumatic cuff, proximally on the desired limb (Sato, 2005; Loenneke et al., 2012). The ischemic nature of BFR-RE causes the accumulation of metabolites in the muscle interstitium, which thereby stimulates muscle growth (Takada et al., 2012).

BFR-RE is widely practiced by bodybuilders and athletes, as it increases muscle strength and mass at low levels of physical exertion. As such, it has recently moved into the clinic as a rehabilitation method, especially for patients incapable of high-intensity exertion and who may otherwise struggle with muscle rehabilitation. The efficacy of BFR-RE in musculoskeletal rehabilitation has been demonstrated (Hughes et al., 2017), and recent interest has focused on the utility of BFR-RE for cardiovascular rehabilitation (Angelopoulos et al., 2023; da Cunha Nascimento et al., 2020; Kambic et al., 2023; Wong et al., 2018). Concerns have been raised regarding the safety of BFR-RE in all populations, citing a potentially increased risk of adverse cardiovascular events induced by activation of the exercise pressor reflex (EPR—muscle metaboreflex and muscle mechanoreflex) secondary to arterial occlusion (Spranger et al., 2015; Cristina-Oliveira et al., 2020). Of particular note are patients with cardiovascular disease who generally have exaggerated EPR responses during exercise (Spranger et al., 2015). While patients with cardiovascular disease could potentially benefit from BFR-RE due to their inability to tolerate high-intensity exercise, a comprehensive understanding of the cardiovascular responses to BFR-RE is crucial to its safe application in the clinic.

## 2 Cardiovascular responses to BFR-RE

Multiple studies have assessed the efficacy of BFR-RE, at both low- and high-intensity (as a percentage of 1RM), in stimulating muscle hypertrophy and increasing muscle strength (Lowery et al., 2014; Pearson and Hussain, 2015; Centner et al., 2019; Grønfeldt et al., 2020; Kataoka et al., 2022), but fewer such studies have assessed the hemodynamic changes with BFR-RE, which would be implicated in any EPR response (Lemos et al., 2022; Pedon et al., 2022). Of the BRF-RE studies that measure hemodynamics, a wide range of cardiovascular responses are reported. This is largely due to the nonstandard nature in which BFR-RE is currently applied (e.g., low-vs. high-load, arm vs leg, type of occlusion device, occlusion pressure, intermittent vs. continuous occlusion). While efforts have been made to integrate the muscle performance-related findings of BFR-RE studies (Pearson and Hussain, 2015; Hughes et al., 2017; Grønfeldt et al., 2020) and create a standard protocol for its application (Patterson et al., 2019), there remains a lack of unification of the cardiovascular responses to BFR-RE. Recent meta-analyses (Lemos et al., 2022; Pedon et al., 2022) have analyzed extant BFR-RE studies in which cardiovascular responses were measured and concluded that BFR-RE does not meaningfully impact the cardiovascular system.

Lemos et al. (2022) compared BFR-RE studies that investigated cardiovascular responses to low-load BFR-RE vs low- and/or high-load unrestricted, or free-flow, resistance exercise (FF-RE). The Authors concluded that low-load BFR-RE elicits blunted cardiac output and heart rate responses when compared to high-load FF-RE. By and large, the studies included in this meta-analysis (*n* = 17) measured cardiovascular responses to BFR-RE during rest and post-exercise (with the cuff deflated). However, two studies included in the analysis measured cardiovascular responses during BFR-RE. Brandner et al. (2015) showed that systolic blood pressure (SBP), diastolic blood pressure (DBP), and heart rate (HR) were significantly elevated during intermittent BFR-RE compared with low-load FF-RE. Similarly, Vieira et al. (2013) reported that SBP, DBP, and HR were significantly greater during BFR-RE when compared to low-intensity FF-RE. While both of these studies are included in the meta-analysis, the aforementioned BFR-RE data *during* exercise were not reported or commented on. This may be attributed to the design of the analysis, in which reportable acute cardiovascular response data are collected pre- and post-exercise.

Pedon et al. (2022) compared BFR-RE studies that investigated cardiovascular responses to low-load BFR-RE vs low- and/or high-load FF-RE. The Authors concluded that there was no difference in cardiovascular responses between low-load BFR-RE and low-load FF-RE. Similar to Lemos et al. (2022), a majority of the studies included in the meta-analysis (12 of 15) measured and compared cardiovascular responses during rest and post-exercise (with the cuff deflated). However, three studies included in the analysis measured cardiovascular responses during BFR-RE. Staunton et al. (2015) measured cardiovascular responses during low-load BFR-RE compared with low-load FF-RE. The study reported that SBP, DBP, and HR were significantly elevated during the final 30 s of exercise (with the blood pressure cuff still inflated). These are the only cardiovascular data *during* BFR-RE included in the meta-analysis. Takano et al. (2005) measured cardiovascular responses during BFR-RE and showed that SBP, DBP, and HR were significantly elevated during low-intensity BFR-RE when compared with low-intensity FF-RE. However, Pedon et al. (2022) only included data comparing peak BFR-RE cardiovascular responses to rest. Finally, Poton and Polito (2016) measured cardiovascular responses during BFR-RE and showed that SBP, DBP, and HR were significantly elevated during set three (out of 3) of low-intensity BFR-RE when compared with low-intensity FF-RE. However, Pedon et al. (2022) only included the first two sets of BFR-RE where there were no significant differences in any hemodynamic parameters.

While the aforementioned meta-analyses concluded that BFR-RE does not meaningfully impact the cardiovascular system, in contrast, a comprehensive systematic analysis investigating the effects of BFR-RE on hemodynamics concluded that low-intensity BFR-RE significantly increases SBP, DBP, and HR when compared to low-intensity FF-RE (Neto et al., 2017). However, it was also concluded that these changes are within the normal range and thus this method may be considered safe and viable for special populations, such as the elderly and cardiac patients. Concerningly, the vast majority of data assessed from these studies were collected pre- and post-exercise.

## 3 The muscle metaboreflex

Caution against widespread implementation of BFR training in the clinic has been raised (Spranger et al., 2015), given that BFR exercise induces the accumulation of muscle metabolites (Takano et al., 2005; Suga et al., 2009), muscle metabolites elicit the muscle metaboreflex (Boushel, 2010), and the EPR augments cardiovascular responses during exercise (Boushel, 2010). By design, Pedon et al. (2022) and Lemos et al. (2022) did not analyze cardiovascular response data to *during* BFR-RE, and therefore these meta-analyses were also unable to assess to what extent, if any, the muscle metaboreflex is engaged during BFR-RE. Thus, the conclusions of these studies that the cardiovascular responses to BFR-RE are either blunted or no different than FF-RE may be misleading.

Importantly, a few studies have collected hemodynamic data *during* BFR-RE that show significantly exaggerated cardiovascular responses (notably SBP, DBP, and HR) when compared with FF-RE (Takano et al., 2005; Brandner et al., 2015; Staunton et al., 2015; Poton and Polito, 2016). Other studies have also reported significantly elevated cardiovascular responses between sets (Downs et al., 2014; Scott et al., 2018; Franz et al., 2020) and immediately following BFR-RE (Barnett et al., 2016; Jessee et al., 2017; Bell et al., 2018; Hori et al., 2020), both during sustained cuff inflation. While post-exercise is distinct from exercise, maintaining arterial occlusion immediately following exercise is a method of isolating the muscle metaboreflex–termed post-exercise muscle ischemia (PEMI) (Spranger et al., 2013). More specifically, muscle metabolites that accumulate during ischemic exercise (such as BFR-RE) elicit the muscle metaboreflex, and if arterial occlusion is sustained following exercise, the metabolites are trapped within the muscle interstitium and thus the reflex remains engaged (Alam and Smirk, 1937). PEMI is traditionally performed with complete arterial occlusion and, during PEMI, arterial blood pressure remains elevated for as long as the ischemia is maintained (Alam and Smirk, 1937). Hori et al. (2020) demonstrated that the muscle metaboreflex is elicited during BFR-RE as SBP was significantly elevated during a suprasystolic PEMI maneuver.

For safety reasons, BFR-RE is generally performed with 40%–80% (Scott et al., 2015) of complete arterial occlusion (i.e., arterial occlusion pressure (AOP)) (Jessee et al., 2016). Therefore, the muscle metaboreflex is unlikely to be fully engaged as metabolites are not completely trapped within the muscle interstitium. Nonetheless, studies have reported significantly elevated SBP between sets (Downs et al., 2014; Scott et al., 2018; Franz et al., 2020) and immediately following a bout of BFR-RE (Barnett et al., 2016; Jessee et al., 2017; Bell et al., 2018; Hori et al., 2020), both during sustained cuff inflation. Both of these settings mimic PEMI and these findings further point to muscle metaboreflex activation during BFR-RE. Supporting this result, a novel technique was developed for approximating SBP during BFR-RE. Immediately post-BFR-RE, cuff occlusion pressure is increased until an AOP is established (given as SBP increases, a higher cuff pressure must be generated to prevent perfusion). Using this approach, studies have demonstrated a significant increase in SBP, expressed as a relative increase in AOP (Barnett et al., 2016; Jessee et al., 2017; Bell et al., 2018). This increase is further exacerbated by increasing initial cuff occlusion pressure or muscle load (Jessee et al., 2017), suggesting this response is correlated with the intensity of BFR-RE.

## 4 Discussion

The majority of studies assessing the cardiovascular responses to BFR-RE fail to address the hemodynamic changes *during* BFR-RE, capturing only pre- and post-exercise data (Lemos et al., 2022; Pedon et al., 2022). In addition, these studies are lacking in standardization of protocol design, especially in the type, intensity, occlusion pressure, time under tension, and limb of exertion. Overall, this has prevented meaningful conclusions regarding the hemodynamic consequences of BFR-RE.

BFR training has been shown to be an effective treatment modality for orthopedic rehabilitation patients (Hughes et al., 2017), paving the way for increased interest in its use in patients with cardiovascular disease (da Cunha Nascimento et al., 2020; Kambic et al., 2023; Wong et al., 2018). While several studies have investigated the utility of BFR exercise in coronary artery disease (Kambič et al., 2019; Kambič et al., 2021) and hypertension (Araujo et al., 2014; Chulvi-Medrano, 2015; Cezar et al., 2016; Pinto and Polito, 2016; Barili et al., 2018), most of these studies only assessed cardiovascular responses pre- and post-BFR exercise, not *during* BFR exercise. For example, Araujo et al. (2014) reported that low-intensity BFR-RE in hypertensive women reduced post-exercise SBP more than moderate-intensity BFR-RE. The study concluded that low-intensity BFR-RE may be a useful therapy for reducing blood pressure in hypertensive individuals. Importantly, the study additionally reported that SBP was substantially higher during BFR-RE when compared with FF-RE. Pinto and Polito (2016) reported exaggerated cardiovascular responses (SBP, DBP, and HR) during low-intensity BFR-RE in hypertensive women compared with high-intensity FF-RE. In a subsequent study, this group reported no differences in cardiovascular responses during low-intensity BFR-RE in hypertensive women compared with high-intensity FF-RE (Pinto et al., 2018). Interestingly, the study reported that SBP and DBP were substantially elevated during the recovery period immediately following BFR-RE when compared with FF-RE, suggesting BFR-RE-induced muscle metaboreflex activation. Finally, a systematic review on the cardiovascular responses to BFR exercise in hypertensive subjects concluded that no definitive recommendation can be made due to the inadequate methodological design of the studies (da Cunha Nascimento et al., 2020). Therefore, the findings and recommendations of these studies should be critically examined.

In summary, while there has been a considerable amount of research on the cardiovascular responses to BFR-RE, most studies have not investigated the cardiovascular responses elicited during exercise with arterial occlusion. The overextension of pre- and post-exercise BFR-RE hemodynamic data as a surrogate for the cardiovascular responses during BFR-RE could result in misleading conclusions. Rest and recovery data cannot be used to establish the safety of BFR-RE, especially for clinically vulnerable populations. A complete understanding of the cardiovascular responses *during* BFR-RE is prerequisite when considering the application of BFR-RE for individuals with cardiovascular disease (e.g., hypertension, heart failure, and peripheral artery disease) who generally exhibit exaggerated muscle metaboreflex and cardiovascular responses during traditional FF-RE (Hammond et al., 2000; Smith et al., 2005; Crisafulli et al., 2007; Leal et al., 2008; Tsuchimochi et al., 2010; Mizuno et al., 2011; Li and Xing, 2012; Muller et al., 2012; Greaney et al., 2014). Thus, future studies should modify and standardize current methodologies to capture the real-time cardiovascular responses to BFR-RE.
